# Virtual reality for opioid reduction in postoperative cardiac and thoracic surgery: A randomized controlled trial protocol

**DOI:** 10.1016/j.mex.2026.104029

**Published:** 2026-07-04

**Authors:** David Duarte de Araujo, Suely Pereira Zeferino, Filomena Regina Barbosa Gomes Galas

**Affiliations:** a Department of Surgery, Anesthesiology, Surgical Sciences and Perioperative Medicine Program (ACCEPT), Faculdade de Medicina da Universidade de São Paulo, Av. Dr. Arnaldo, 455, São Paulo, 01246-903, Brazil; b Heart Institute (InCor), Hospital das Clinicas HCFMUSP, São Paulo, Brazil

**Keywords:** Digital therapeutics, Distraction therapy, Pain management, Immersive technology, Enhanced recovery after surgery (ERAS)

## Abstract

This protocol describes a randomized controlled trial designed to evaluate the efficacy of Virtual Reality (VR) as a non-pharmacological intervention for pain management in the immediate postoperative period of cardiac and thoracic surgeries. Postoperative pain in these major procedures is often intense and historically managed with high doses of opioids, which are associated with significant side effects and potential dependence. By leveraging the "limited capacity theory of attention," this study uses immersive and passive VR environments to compete with painful stimuli for cognitive resources, thereby aiming to reduce total opioid consumption.

• This protocol establishes a standardized three-arm methodology to compare active VR immersion, passive VR contemplation, and conventional multimodal analgesia in a high-complexity surgical setting.

• It provides a detailed framework for integrating digital distraction technology into intensive care unit (ICU) routines, including a rigorous equipment hygiene protocol to ensure patient safety.

• The study focuses on a specific younger demographic (16–40 years) to evaluate the impact of VR on recovery outcomes, opioid sparing, and patient satisfaction.

## Graphical abstract

**Figure:**
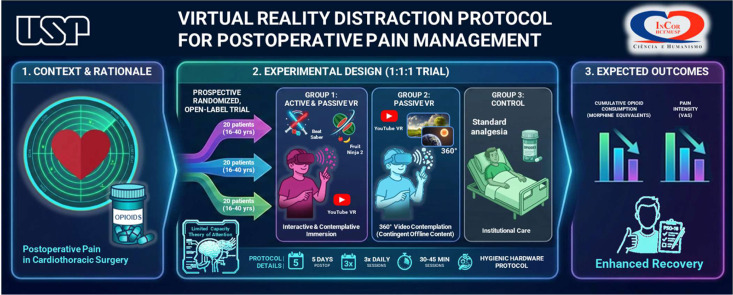

## Specifications table

**Table:** 

**Subject area**	Medicine and Dentistry
**More specific subject area**	Anesthesiology and Perioperative Medicine.
**Name of your protocol**	Virtual Reality Distraction Protocol for Postoperative Pain Management.
**Reagents/tools**	VR Headsets (Meta Quest), Fruit Ninja 2, Oculus First Contact, Beat Saber, First Hand, YouTube VR
**Experimental design**	Prospective, randomized, open-label, three-arm clinical trial (1:1:1 ratio).
**Trial registration**	ClinicalTrials.gov, NCT07063394
**Ethics**	The study protocol was approved by the Research Ethics Committee of the Hospital das Clínicas da Faculdade de Medicina da Universidade de São Paulo (CAPPESQ - HC-FMUSP). All participants will be informed about the study's objectives, procedures, potential risks, and benefits. We confirm that the relevant written informed consent (Termo de Consentimento Livre e Esclarecido - TCLE) will be obtained from all subjects prior to their inclusion in the study, ensuring the protection of their autonomy and dignity.
**Value of the Protocol**	• This protocol establishes a structured framework for using Virtual Reality (VR) as a low-cost, non-pharmacological intervention aimed at reducing opioid consumption in high-complexity surgical patients. • The study provides a detailed guide for implementing digital distraction in an Intensive Care Unit (ICU) setting, including a rigorous hardware hygiene protocol to ensure patient safety and clinical feasibility. • By comparing active game immersion with passive 360° video contemplation, the protocol helps identify which cognitive distraction mechanisms are most effective in modulating acute postoperative pain.

## Background

The management of postoperative pain in cardiothoracic surgery remains a critical challenge, as inadequate analgesia more than doubles the risk of postoperative complications (OR 2.56), including respiratory dysfunction and prolonged hospitalization [1]. Furthermore, while opioids represent the analgesic cornerstone, their dose-dependent administration induces adverse events in approximately 20% of patients, precipitating significant morbidity such as respiratory depression, emesis, and long-term dependence [2].

This proposed protocol addresses the clinical need for innovative, non-pharmacological adjuncts to multimodal analgesia. Grounded in the limited capacity theory of attention, highly immersive distractors exhaust finite cognitive resources, modulating shared cortical networks [3] and triggering neurochemical responses, such as endorphin release, to effectively reduce perceived pain intensity [4].

Virtual Reality (VR) provides a superior level of immersion compared to traditional distraction methods, yielding up to an 88% decrease in postoperative pain [5]. Therefore, this study is designed as a proof-of-concept efficacy trial to standardize VR application in the Intensive Care Unit (ICU) setting, targeting patients (16–40 years old) undergoing cardiothoracic surgery. Our primary objective is to evaluate the preliminary clinical efficacy of this multi-session VR protocol on cumulative opioid consumption and pain intensity. It distinguishes itself by evaluating two digital strategies—active gaming, associated with the attenuation of cortical pain-related amplitudes [6], and passive 360° video contemplation—to determine their respective impact on postoperative recovery.

By establishing this early-stage framework, this protocol provides researchers and clinicians with a reproducible model for integrating digital health technology into the perioperative period, emphasizing technical feasibility, equipment hygiene, and patient safety, while gathering preliminary efficacy signals to guide future definitive trials.

## Study design and randomization

The VR-USP study follows a Prospective Randomized Open, Blinded Endpoint (PROBE) three-arm clinical trial design with a 1:1:1 allocation ratio. The trial protocol was reviewed and approved by the Institutional Review Board (Protocol Number: CAAE 74,181,523.0.0000.0068) and prospectively registered in a primary clinical trial registry (Registry Number: NCT07063394). To evaluate the intervention’s robustness within a diverse cardiothoracic population, a block randomization sequence, consisting of twenty fixed blocks of three, is generated by an independent statistician utilizing Microsoft Excel software. This pragmatic approach is selected to reflect real-world clinical heterogeneity, ensuring that the trial’s findings maintain high external validity for routine intensive care workflows. Allocation concealment is ensured through the use of sequentially numbered, opaque, and tamper-evident sealed envelopes, numbered from 1 to 60. These envelopes are stored in a locked cabinet controlled by an independent clinical nurse who has no contact with the trial participants. Furthermore, the specific envelope for each participant is only released to the research team after a designated investigator obtains the appropriate consent documents, including parental consent and minor assent for patients under 18 years of age, and enrolls the patient. Consequently, the recruiters remain blinded to the allocation sequence prior to inclusion, and the assigned intervention arm is revealed only post-enrollment, thereby maintaining appropriate allocation concealment.

## Standard of care framework

Irrespective of group allocation, all participants receive the institutional standard multimodal anesthesia and analgesia protocol, which is managed by the Acute Pain Service. Intraoperatively, all patients undergo a standardized general anesthesia regimen, including continuous hemodynamic monitoring (5-lead electrocardiogram, pulse oximetry, non-invasive and invasive arterial blood pressure) and controlled mechanical ventilation. Crucially, to provide preventive analgesia and mitigate the neuroendocrine stress response associated with major surgical trauma, early analgesic interventions—incorporating regional techniques such as thoracic epidural placement (T5-T12) or fascial plane blocks, tailored to the specific surgical approach—are systematically performed immediately following anesthetic induction and prior to the surgical incision. Postoperatively, this regimen employs a balanced strategy integrating peripheral analgesics, nonsteroidal anti-inflammatory drugs, and targeted opioid administration. In the immediate postoperative phase, pain is controlled through intravenous morphine titration, consisting of 2 mg boluses administered every 5 to 10 min until a Visual Analogue Scale score below 3 is achieved.

Subsequently, analgesic maintenance is provided via Patient-Controlled Analgesia pumps. These devices utilize a standardized 0.5 mg/ml morphine solution, programmed for demand boluses of 0.5 to 1.0 mg with a 7 to 15 min lockout interval. Furthermore, pain intensity is systematically evaluated as a vital sign during routine hemodynamic checks utilizing the Visual Analogue Scale (VAS). Concurrently, the Richmond Agitation-Sedation Scale (RASS) is monitored by the ICU clinical team as a safety and eligibility parameter. To ensure protocol fidelity and patient safety, a Virtual Reality session is only initiated if the patient is in an optimal state of alertness, defined as a RASS score between −1 (drowsy) and +1 (restless). Patients presenting with severe agitation (RASS ≥ +2) or deep sedation (RASS ≤ −2) will have their sessions postponed until clinical stabilization. Consequently, the uniform application of this baseline framework across all study arms ensures that any variations in cumulative opioid consumption can be attributed directly to the digital interventions being investigated.

## Sample size and study population

The fundamental rationale relies on the limited capacity theory of attention, positing that highly immersive multisensory environments exhaust finite cognitive resources to mitigate acute pain perception [7]. Furthermore, as demonstrated by Lier et al., virtual reality yields significant overall analgesic efficacy (SMD = −0.65), with particularly robust effects observed among younger demographics [8]. Consequently, the trial enrollment focuses on 60 patients aged 16 to 40 years. Eligible procedures include cardiac and specific thoracic surgeries, such as pulmonary decortication, video-assisted thoracoscopic surgery (VATS), lung biopsy, and Nuss bar pectus excavatum repair.

As this trial is designed as an early-stage, proof-of-concept efficacy study in the specific context of cardiac surgery, a formal a priori power calculation for clinical efficacy is not performed. Instead, a pragmatic sample size of 20 patients per arm (total N = 60) is established. This sample size is aligned with phase IIa / proof-of-concept trials aimed at detecting large clinical effect sizes, evaluating the safety profile of the intervention, and providing initial estimates of variance (mean and standard deviation) to adequately power future, large-scale definitive multicenter trials.

While previous studies have demonstrated substantial effect sizes for VR analgesia in other surgical contexts, extrapolating those parameters to a three-arm, highly invasive cardiothoracic population is not methodologically sound for an a priori power calculation. Therefore, this sample size of 60 serves as a pragmatic and methodologically sound approach. It is sufficiently large to evaluate our primary feasibility metrics and safety profiles, while providing exploratory signals regarding the cumulative opioid consumption to inform future research.

The inclusion criteria encompass patients aged 16 to 40 years, a demographic targeted as meta-analyses confirm that younger individuals exhibit significantly greater analgesic efficacy from virtual reality interventions [8]. Eligible surgical procedures comprise specific cardiac interventions, including valve surgery, myocardial revascularization, aortic procedures, and congenital heart defect corrections [9], alongside thoracic operations, namely pulmonary decortication, video-assisted thoracoscopic surgery (VATS) [10], lung biopsy, and Nuss bar pectus excavatum repair. The aggregated analysis of these major cardiothoracic procedures is scientifically justified by their shared pathophysiological pathways and homogeneous moderate-to-high postoperative pain intensity, which is consistently exacerbated by surgical chest wall trauma, inherent to both sternotomies [11] and thoracoscopic ports [12], as well as the nociceptive stimulus of thoracic drains [13]. Furthermore, because pain perception is fundamentally constructed by a central neural network independently of the specific peripheral incision [14], virtual reality effectively acts as an immersive cognitive distractor that attenuates pain-related cortical processing; thus, its therapeutic efficacy remains robust and comparable across distinct surgical traumas [15]. This unified approach is further supported by prospective evidence demonstrating that the incidence and intensity of acute postoperative pain do not significantly differ between patients undergoing median sternotomy and those undergoing thoracic incisions [16]. Finally, a preoperative Mini-Mental State Examination (MMSE) score of 25 or higher remains a mandatory inclusion criterion for all three study arms. By enforcing this universal cognitive threshold, the study ensures that participants in both the intervention and control groups possess the necessary cognitive capacity for effective interaction with immersive virtual reality environments [17], reliable participation in pain assessment protocols, and a clear understanding of the study instructions.

Conversely, the exclusion criteria are systematically applied and scientifically justified to ensure patient safety and protocol fidelity:

• **Clinical and Operational Status:** Patients currently requiring invasive mechanical ventilation at the time of the scheduled intervention (due to physical interference with the VR headset and inability to interact), or those presenting with active infectious-contagious diseases. Recent surgical trials dictate that these exclusions are critical, as such clinical states limit the required active cooperation, pose risks of device displacement, and significantly increase the likelihood of cross-contamination with head-mounted displays [18].
• **Cognitive and Neurological Baseline:** Patients with a documented cognitive deficiency, defined by a Mini-Mental State Examination (MMSE) score of 24 or lower, as well as those diagnosed with other cognitive disorders or spatial orientation deficiency. This specific cutoff is supported by Cochrane systematic reviews, which demonstrate that an MMSE score of 24 provides an optimal balance of 85% sensitivity and 90% specificity for detecting cognitive impairment, thereby preventing the inclusion of individuals unable to fully comprehend instructions or interact with complex virtual environments [19].
• **Physical and Sensory Constraints:** Individuals with sensory or physical limitations, specifically severe visual impairments and upper limb motor deficits. This exclusion guarantees that patients possess the physical ability to safely interact with the hardware and fully perceive the virtual environment.
• **Psychological and Vestibular Intolerance:** Conditions that directly contraindicate immersive technologies, notably claustrophobia and motion sickness, including labyrinthine diseases. While recent meta-analyses confirm that virtual reality is generally safe, mild adverse events such as nausea, dizziness, and cybersickness are still reported, with some clinical trials noting dropout rates of up to 13% due to these symptoms, making the exclusion of highly susceptible populations essential to guarantee patient safety and maintain strict protocol adherence [20].

## Virtual reality interventions

For the intervention arms, a progressive additive model is applied to ensure clear methodological differentiation. Group 3 serves as the control and receives the institutional standard multimodal anesthesia and analgesia protocol. To maintain high external validity and reflect real-world intensive care conditions, pharmacological management and rescue analgesia for all groups are strictly governed by the institutional Standard Operating Procedure for Acute Pain Management. According to this protocol, pain is objectively assessed during every routine vital sign check. Adequate pain control is institutionally defined as a Visual Analogue Scale (VAS) score of 3 or less. Consequently, subjective bedside dosing is minimized, as any reported pain score of VAS ≥ 4 (categorized as moderate to severe pain) triggers mandatory re-evaluation and the administration of standardized rescue interventions—including oral, intermittent intravenous, or PCA titrations—until the target of VAS < 3 is achieved. Furthermore, to preserve the pragmatic nature of the trial, participants in the control group are not restricted from using standard non-immersive personal devices, such as smartphones or hospital televisions. Restricting access to these devices is considered ethically and logistically inviable due to the essential need for family communication during a critical postoperative period and the inherent setup of shared hospital rooms. Therefore, this study is pragmatically designed to evaluate the supplementary efficacy of virtual reality immersion against the baseline noise and unstructured digital distractions that already constitute the real-world standard of care. This methodological allowance is further grounded in the premise that the therapeutic mechanism of virtual reality relies on multisensory immersion, which fundamentally differs from the limited cognitive engagement provided by conventional two-dimensional screens.

Regarding the experimental arms, Group 2, designated as the passive therapy arm, receives the identical standard clinical protocol supplemented with the Meta Quest VR headsets, providing access to the YouTube VR platform for passive immersion. Crucially, rather than restricting individuals to a standardized preselected playlist, the protocol allows the free selection of 360-degree videos, encompassing nature, space, or cultural events, based on individual preferences. This patient-centered design is methodologically grounded in the premise that cognitive distraction, which is the primary mechanism of action for pain modulation, is maximized when the visual stimulus aligns with the patient's intrinsic interests, thereby ensuring deeper emotional engagement.

Consequently, Group 1 represents the unrestricted enriched therapy arm, receiving the standard clinical protocol along with VR headsets equipped with both the passive YouTube VR content and immersive interactive gaming experiences. These active applications include Beat Saber, First Hand, Fruit Ninja 2, and Oculus First Contact, selected to ensure a diverse range of motor and cognitive engagement. By intentionally allowing free transition between active and passive content, this pragmatic design aims to evaluate whether providing the additional option of interactivity yields superior clinical outcomes and higher protocol adherence compared to the strictly passive device of Group 2. Rather than enforcing a rigid mechanistic isolation of active versus passive modalities—which could induce fatigue or jeopardize safety in acutely debilitated postoperative patients—this hybrid approach accommodates the fluctuating physical capacity inherent to intensive care recovery. Patients can safely initiate therapy with passive contemplation and progressively scale their physical and cognitive engagement as their clinical status improves. Under this framework, the primary intervention metric remains defined by the total duration of hardware exposure, reflecting the cumulative dose of immersive cognitive distraction driven by patient autonomy, regardless of the specific software application selected.

## Trial procedures and data collection

The protocol begins in the preoperative phase with a systematic cognitive screening applied to all potential participants. Specifically, the Mini-Mental State Examination (MMSE) is administered to every patient prior to surgery, including those subsequently randomized to the control group, to ensure a baseline of cognitive function that is homogeneous across the entire cohort. Furthermore, once clinically stabilized after surgery and extubated in the ICU, randomized patients start their assigned VR sessions from the first to the fifth postoperative day. The target duration for each session is 45 min to maximize the immersive analgesic effect. However, to prioritize patient comfort and mitigate visual fatigue, the protocol permits a voluntary early cessation at 30 min based on spontaneous patient requests, thereby defining the 30 to 45 min therapeutic window. These sessions are performed three times daily. The total cumulative time of hardware exposure is recorded for each participant to ensure a standardized evaluation of the immersive therapeutic dose across all intervention groups. Specifically, this therapeutic dose will be analyzed as a continuous variable, calculated by summing the total minutes across all 15 sessions. Early interruptions are not classified as protocol deviations, but rather as expected natural fluctuations in patient tolerance, being appropriately captured as a lower cumulative dose within the Intention-to-Treat (ITT) framework.

Researchers provide a training phase to ensure the patient is comfortable with the hardware and the virtual environment. Within this framework, the voluntary acceptance of the informed consent form serves as a natural filtration mechanism for technological affinity; by enrolling only participants who express interest and comfort with immersive digital activities, the study ensures a cohort predisposed to optimal therapeutic engagement, thereby mitigating potential variance related to prior gaming experience. Furthermore, to guarantee methodological consistency and eliminate behavioral bias, all research personnel undergo a standardized training program in accordance with Good Clinical Practice guidelines. The recruitment team, responsible for administering the informed consent form, is trained on the study rationale and experiences the virtual reality therapy to ensure standardized and neutral patient communication. Similarly, the bedside clinical team, tasked with conducting the sessions and collecting hemodynamic data, receives formal instruction emphasizing the critical importance of measurement accuracy. These professionals also complete hands-on simulation modules with the specific applications to assist patients with hardware operation. Finally, the data management team is trained on data extraction from both the institutional electronic health record system (Si3) and the bedside case report forms detailing the specific events of each therapeutic session. This rigorous training ensures the standardized transposition of all clinical variables into the REDCap platform, guaranteeing fidelity and traceability in data handling.

Data collection begins upon enrollment, encompassing demographic variables, biometric indices, and baseline clinical risk stratification. Specifically, the American Society of Anesthesiologists physical status classification will be assessed for the entire cohort, while the EuroSCORE II will be calculated for patients undergoing cardiac procedures. Preoperative cognitive function is quantified using the Mini Mental State Examination. Additionally, intraoperative parameters, including cardiopulmonary bypass duration, total anesthesia time, and intraoperative opioid titration, are recorded to account for potential confounding variables in the postoperative analgesic response. To ensure maximum data integrity and adherence to good clinical practice guidelines, all gathered clinical information will be securely entered and managed using the Research Electronic Data Capture (REDCap) electronic data capture tools hosted at the institution.

## Clinical endpoints and blinding

The primary clinical endpoint is defined as the cumulative morphine milligram equivalents consumed postoperatively, which is objectively quantified throughout the entire evaluation period. This fundamental outcome is monitored by tracking patient-controlled analgesia pump dosing logs to accurately record both requested and administered boluses. By assessing total opioid consumption within a pragmatic clinical framework, the study captures the intervention’s efficacy as an adjunct to routine postoperative analgesic management, accounting for the inherent clinical variability of an intensive care setting. Furthermore, due to the randomized design, it is assumed that exposure to institutional sedation protocols, chest physiotherapy, and intensive care environmental noise will be homogeneously distributed across all three arms, thereby neutralizing the influence of these confounding factors when comparing the intervention and control groups. To ensure accurate comparative analysis, all administered opioids, including rescue doses of medications such as tramadol, will be converted into cumulative morphine milligram equivalents according to standardized international pharmacological guidelines. Concurrently, the total daily consumption of non-opioid rescue analgesics, specifically dipyrone and paracetamol, will be independently calculated and recorded as the total cumulative daily dose in milligrams.

In parallel, as a crucial secondary clinical outcome, pain intensity is measured using the Visual Analogue Scale (VAS) at rest and during movement, which is specifically elicited by instructing the patient to take a deep breath. Evaluating dynamic pain represents the clinical gold standard, as joint practice guidelines from the American Pain Society and the American Society of Anesthesiologists emphasize that assessing pain during movement or activity is essential to accurately capture postoperative discomfort and guide functional recovery [21]. These VAS assessments are conducted at predefined and standardized time points throughout the day, ensuring temporal consistency across all three study arms. To clarify the Prospective Randomized Open, Blinded Endpoint (PROBE) design, we have explicitly defined the blinding status of all involved personnel. Due to the physical and highly visible nature of the Virtual Reality headsets, complete double-blinding is structurally impossible. Therefore, to mitigate detection and performance bias, a strict blinding matrix is established (Table 1).

**Table 1:** Blinding matrix for the PROBE design.

Role	Blinding Status	Justification / Mitigation Strategy
Participants	Unblinded	Inherent to the use of highly visible VR equipment.
VR Application Team	Unblinded	Researchers responsible for device setup and safety monitoring.
ICU Clinical Team	Unblinded	Cannot be blinded due to the shared, open ICU environment.
Pain Outcome Assessors	Blinded	Distinct staff exclusively assessing VAS. Patients are instructed daily not to discuss VR.
Statistician	Blinded	Data delivered with masked group codes (e.g., Group A, B, C).

By completely separating the personnel who administer the VR sessions from the clinical professionals who assess the Visual Analogue Scale (VAS) scores, the outcome assessors remain blinded to the assigned intervention. Additionally, the primary endpoint (cumulative opioid consumption) is quantified objectively via automated PCA pump logs and standardized medical records, structurally preventing expectancy bias from the outcome assessors.

Alongside these daily clinical assessments, the safety and tolerability of the intervention are continuously evaluated. Hemodynamic and respiratory parameters, comprising heart rate, mean arterial pressure, oxygen saturation, and respiratory rate, are recorded immediately before and after each virtual reality session. The research team actively monitors for signs of cybersickness, such as nausea or dizziness, as well as physical adverse events ranging from neck pain to skin lesions caused by the headset. Consequently, safety and hygiene protocols, which have been approved by the Hospital Infection Control Committee (CCIH), are paramount for trial replication. The equipment undergoes a cleaning process involving the disinfection of the headset's external hard plastic surfaces with 70 percent isopropyl alcohol wipes. To preserve structural integrity and prevent degradation of the anti-reflective coatings, the VR optical lenses and the auxiliary mirroring tablet screens are cleaned using a specific non-alcoholic optical screen cleaning solution (Implastec, Brazil) applied with dry microfiber cloths. To prevent cross-contamination, patients are provided with specific disposable ocular masks designed for virtual reality headsets. Furthermore, the head straps are wrapped in a protective plastic film that is replaced between each patient use. Throughout the process, the protocol allows for immediate cessation of the session if any discomfort arises. Moreover, in the unlikely event of a serious or unanticipated adverse reaction, the research team is mandated to halt the intervention immediately, provide appropriate medical care, and report the incident to the Institutional Review Board within 24 h, in compliance with ethical guidelines.

Furthermore, broader secondary clinical trajectories and major postoperative complications are monitored to provide a comprehensive recovery profile. The study tracks the length of stay in both the intensive care unit (ICU) and the hospital, the total cumulative duration of mechanical or noninvasive ventilation required throughout the ICU stay, and the incidence of critical events such as delirium, assessed via the CAM ICU protocol, or paralytic ileus.

Finally, to evaluate the overall hospital experience, patient satisfaction is quantified using the short-form 18-item Patient Satisfaction Questionnaire (PSQ 18) on the fifth postoperative day or prior to hospital discharge. Surpassing the exposure time typically evaluated in previous trials, this protocol delivers a cumulative immersion dose of up to 11.25 h over five days (45 min, three times daily). Consequently, this comprehensive instrument is selected because this sustained and highly immersive acute pain management is hypothesized to generate a robust halo effect. This extensive exposure is theorized to positively alter the patient's global perception of the entire hospital stay, thereby allowing the reliable measurement of core dimensions of care, including technical quality and general satisfaction [22].

## Statistical analysis plan

The statistical analysis plan will be executed using SPSS software. Initially, continuous variables will be checked for normal distribution using the Shapiro-Wilk test. To compare baseline characteristics and cross-sectional continuous data among the three distinct groups, a One-Way ANOVA or Kruskal-Wallis test will be utilized, as appropriate. Furthermore, categorical baseline outcomes will be assessed through Chi-squared or Fisher's exact tests.

To account for unequal observation windows arising from early hospital discharge or varying ICU lengths of stay, the primary endpoint will be time-standardized. The absolute cumulative opioid consumption will be divided by the individual patient's exact observation time, yielding a standardized rate of Morphine Milligram Equivalents (MME) per 24 h. This primary outcome, along with the longitudinal secondary clinical outcomes (specifically the repeated Visual Analogue Scale scores evaluated at multiple time points), will be analyzed exclusively using Generalized Linear Mixed Models (GLMM). This advanced modeling accounts for intra-subject correlation over time. Crucially, to adjust for the inherent clinical heterogeneity between distinct surgical profiles (e.g., median sternotomy versus thoracic procedures like VATS or Nuss repair), the 'Surgical Type' will be included as a fixed-effect covariate in the model.

To preserve the integrity of the initial randomization sequence while avoiding the imputation of an entire evaluation period for early dropouts, the primary efficacy analysis will be conducted adhering to a modified Intention-to-Treat (mITT) principle. This approach encompasses all randomized participants who received the allocated intervention and underwent at least one post-randomization clinical assessment. To handle potential missing longitudinal data resulting from early clinical discharge, severe postoperative complications, or protocol non-adherence, the analysis will rely on the inherent capacity of the GLMM to manage missing observations under the Missing at Random (MAR) assumption. Additionally, to account for data that may be Missing Not at Random (MNAR)—particularly due to asymmetric dropout rates driven by intervention-related adverse effects such as cybersickness—a sensitivity analysis utilizing Pattern-Mixture Models will be conducted to assess the robustness of the primary findings. Furthermore, a secondary per-protocol analysis will be performed for participants who completed a minimum of two therapeutic sessions per day to evaluate the intervention’s efficacy under optimal adherence conditions. A standard significance level of p < 0.05 will be established. Finally, the statistician performing the final data analysis will be blinded to the group allocation codes.

## Safety protocols and physical restrictions

To ensure the physical safety of patients recovering from highly invasive cardiothoracic procedures—who present with median sternotomies, chest tubes, and central venous catheters—strict safety protocols are implemented during all interventions. A trained healthcare professional remains continuously at the bedside for the entire duration of every VR session to supervise the patient, manage the hardware, and prevent any hazardous movements. Patients are comfortably positioned in their ICU beds, and upper limb movements are strictly monitored to adhere to standard sternal precautions, preventing excessive shoulder abduction or overextension.

Recognizing the varying physical demands of the software, the active VR arm employs a progressive interaction strategy. Patients are allowed to begin with passive observation or low-intensity applications (e.g., Oculus First Contact). As they demonstrate comfort and clinical stability, they may gradually advance to games requiring moderate arm movement (e.g., Fruit Ninja, Beat Saber), strictly within their pain-free range of motion. This stepwise approach maximizes patient adherence while ensuring physical safety. The preliminary pre-trial pilot phase of this study explicitly tested and validated this supervised, progressive methodology, confirming its clinical safety and operational feasibility without compromising surgical incisions or indwelling lines.

## Protocol validation

The validation of this protocol framework is based on a preliminary, pre-trial pilot phase conducted at the Heart Institute (InCor) with an initial sample of 3 patients, encompassing a total of 45 sessions. This preliminary phase was designed to ensure that the integration of Virtual Reality (VR) technology does not interfere with standard Intensive Care Unit (ICU) monitoring or clinical workflows. This strict preliminary evaluation of technical parameter settings and hardware feasibility aligns perfectly with recent methodological benchmarking for VR pilot designs in thoracic populations, as demonstrated by Cao et al. (2025) [10]. Preliminary technical tests confirmed that the Meta Quest headsets maintain optimal performance for the full duration of the 30 to 45 min sessions, without any instances of overheating or software crashes. Furthermore, during the pilot phase, hospital wireless network blind spots were identified, which initially hindered the functionality of the passive therapy arm that relied heavily on YouTube VR content. To successfully circumvent this technical barrier, high-quality 360-degree videos were downloaded and stored directly on the internal memory of the headsets, ensuring uninterrupted passive therapy delivery even in locations without internet access.

In addition to technical stability, the hygiene protocol using 70 percent isopropyl alcohol and neutral soap was rigorously tested for its impact on equipment durability. Results from the pilot evaluations showed no degradation of the lenses or facial pads, thus validating the long term feasibility of shared device use within a highly regulated hospital setting.

Consequently, patient safety was quantitatively validated throughout the entire pilot period by continuously monitoring vital signs, including heart rate, mean arterial pressure, oxygen saturation, and respiratory rate, alongside tracking signs of cybersickness. In these preliminary assessments, patients within the targeted age group of 16 to 40 years reported high levels of comfort and ease of use with the hand tracking features in the First Hand and Oculus First Contact applications. Across the 45 executed sessions, the incidence of adverse events was limited to a single report of mild dizziness. While this represents an occurrence rate of 33.3% at the patient level (1 out of 3 patients), when analyzed by total therapeutic exposure, the adverse event occurred in only 1 out of the 45 sessions (a 2.2% per-session rate). Although this preliminary sample is too small to draw definitive conclusions regarding overall clinical safety, these pilot results confirmed the initial technical feasibility and general tolerability of the repeated-exposure protocol, allowing the main trial to proceed.

Moreover, the adherence rate was formally assessed, demonstrating that all the participants were able to successfully complete at least two of the three daily sessions, thereby meeting the minimum threshold established for inclusion in the final data analysis.

Finally, the data collection infrastructure was validated through a mock entry process using the REDCap platform. This procedure ensured that all clinical variables, particularly the Visual Analogue Scale (VAS) scores and precise opioid dosages extracted from patient-controlled analgesia (PCA) pumps, are accurately captured, synchronized, and ready for subsequent statistical analysis.

## Limitations

A primary limitation of this protocol is the inability to perform a double-blind study due to the nature of the intervention, as both the participants and the research team are inherently aware of the use of Virtual Reality (VR) headsets. Consequently, this lack of blinding may introduce a performance bias in the subjective reporting of pain levels through the Visual Analogue Scale (VAS) and in the evaluation of patient satisfaction via the PSQ 18 questionnaire. Additionally, patients allocated to the standard care control group might experience resentful demoralization. Knowing they are excluded from the novel technological intervention could negatively influence their subjective psychological state and artificially lower their satisfaction scores.

In addition, a notable limitation inherent to this study design is the presence of attention and expectancy biases. Patients allocated to the VR intervention arms inherently receive more direct, continuous attention from the research team during the setup and supervision of the sessions compared to the standard care control group. This discrepancy in social interaction, combined with the novelty effect of using an immersive technological device, may generate a positive expectancy or placebo effect that independently contributes to pain reduction and anxiety relief. While the use of an objective primary endpoint—cumulative opioid consumption, rigorously calculated by tracking both automated PCA pump logs and electronic medical records (encompassing oral and intermittent intravenous doses)—helps mitigate the subjectivity of self-reported pain scores (VAS), the psychological impact of receiving specialized, dedicated attention cannot be entirely isolated from the specific analgesic effects of the VR therapy itself.

In addition, environmental factors in the Intensive Care Unit, such as excessive noise, frequent clinical interruptions, or the presence of multiple invasive lines and chest tubes, may also severely limit the degree of immersion achieved by the patient.

Moreover, the implementation of this protocol relies on a stable technological infrastructure. As identified during pilot assessments, hospital wireless network blind spots can disrupt the delivery of streaming content, necessitating the use of pre-downloaded offline media as a mandatory contingency for the passive therapy arm. Parallel to these technological barriers, language can act as an immersion limiting factor. Since the primary interactive applications utilized, such as First Hand and Oculus First Contact, feature English interfaces and audio, non-fluent patients might experience a reduced sense of presence. The risk of cybersickness, although minimized by the careful selection of appropriate software, also remains a physiological factor that could lead to the premature cessation of sessions in highly susceptible individuals.

Because the protocol strategically focuses on a specific age group of 16 to 40 years to ensure technological familiarity, the results may not be directly generalizable to older populations, who might face completely different cognitive or physical challenges when interacting with VR technology. Ultimately, as a single center trial conducted within a highly specialized academic institution, the findings may have limited external validity when extrapolated to community hospitals with different resource constraints. Furthermore, the study design restricts the evaluation window to the acute perioperative phase, concluding on the fifth postoperative day or at hospital discharge. This timeframe inherently limits the ability to assess the long term impact of the VR intervention on the prevention of chronic postsurgical pain or prolonged opioid dependence.

Finally, as a proof-of-concept trial, this study has inherent statistical limitations. The pragmatic sample size of 20 patients per arm (N = 60 total) is prospectively established without a population-specific a priori power calculation. Consequently, while this sample size is mathematically adequate to detect large clinical effect sizes and provide initial estimates of variance, the study may be underpowered to identify small-to-moderate differences in cumulative opioid consumption or pain intensity. Therefore, the risk of a Type II error cannot be completely ruled out, and these findings should be interpreted as preliminary efficacy signals that require validation in future, fully powered definitive multicenter trials.

## CRediT author statement

David Duarte de Araujo: Investigation, Methodology, Data Curation, Writing - Original Draft.

Suely Pereira Zeferino: Methodology, Validation, Project administration, Resources.

Filomena Regina Barbosa Gomes Galas: Conceptualization, Supervision, Funding acquisition, Project administration, Writing - Review and Editing.

## Related research article

**For a published article:**

None.

## Declaration of competing interest

The authors declare that they have no known competing financial interests or personal relationships that could have appeared to influence the work reported in this paper.

## Data Availability

No data was used for the research described in the article.
